# Effect of the Marine Polyketide Plocabulin on Tumor Progression

**DOI:** 10.3390/md21010038

**Published:** 2022-12-31

**Authors:** Eleonora Turrini, Francesca Maffei, Carmela Fimognari

**Affiliations:** Department for Life Quality Studies, University of Bologna—C.so d’Augusto, 237, 47921 Rimini, Italy

**Keywords:** marine sponge, plocabulin (PM060184), polyketide, anticancer drugs, antitubulin activity, antiangiogenic activity

## Abstract

Marine sponges represent one of the richest sources of natural marine compounds with anticancer potential. Plocabulin (PM060184), a polyketide originally isolated from the sponge *Lithoplocamia lithistoides*, elicits its main anticancer properties binding tubulin, which still represents one of the most important targets for anticancer drugs. Plocabulin showed potent antitumor activity, in both in vitro and in vivo models of different types of cancers, mediated not only by its antitubulin activity, but also by its ability to block endothelial cell migration and invasion. The objective of this review is to offer a description of plocabulin’s mechanisms of action, with special emphasis on the antiangiogenic signals and the latest progress on its development as an anticancer agent.

## 1. Introduction

The activation of invasion and metastasis is classified by Hanahan and Weinberg as a hallmark of cancer [1]. Indeed, the invasion of cancer cells to nearby tissue and their seeding at distant sites to form metastases remains a central features of cancer malignancies, responsible for 90% of cancer deaths [2]. At the time of cancer diagnosis, at least half of the patients already present with clinically detectable secondary or tertiary foci of tumors, and metastasis can be defined as the most life-threatening event in cancer patients. The metastatic cascade requires three main processes: invasion, intravasation, and extravasation [3]. Changes in cell–cell and cell–matrix adhesion enable cells to dissociate from the primary tumor and invade the surrounding stroma, respectively. To spread, tumor must also initialize the process of angiogenesis, which allows for the transportation of nutrients and the removal of waste products from the tumor site. Thus, angiogenesis allows for tumor development when the mass grows more than 2 mm in diameter [3]. Moreover, blood vessels can provide a route for detached cells to metastasize to distant sites from the primary tumor, a process named intravasation. Once the tumor cells begin to interact with the endothelial cells and penetrate the endothelium and basement membrane, the process of extravasation occurs, and tumors cells start to proliferate and give rise to a secondary tumor [3].

Metastasis is a complex challenge that requires more than one therapeutic agent for effective inhibition. In the current armamentarium to fight cancer, anti-invasive and anti-metastatic drugs are missing [4]. Therefore, embracing the combination therapy model and/or targeting multiple pathways simultaneously seems to be the only strategy to counteract cancer in the final stages of progression, i.e., invasion and metastasis [2].

Natural products have long represented a rich reservoir of bioactive compounds with therapeutic potential, or lead compounds for synthetic optimization [5]. Approximately 80% of the approved chemotherapeutic drugs are based on bioactive natural products. The vast biodiversity of the marine environment, together with the recent improvement in the techniques and analytical methods for the exploration of the deeper sea, increased the number of new molecules from marine origin, which will enrich the anticancer clinical pipeline in the near future [6].

Marine sponges represent an attractive source for new anticancer agents due to their production of a high variety of molecules affecting different cellular and molecular events involved in cancer development [7]. Almost 50 years after the marine sponge obtained cytarabine approval for cancer treatment, other marine drugs have been authorized, such as the tunicate derivative trabectedin, the simplified synthetic sponge derivative eribulin mesylate, and the CD30-targeted antibody drug conjugate brentuximab vedotin, developed from the sea hare pentapeptide dolastatin 10. To date, the marine-derived metabolites under investigation as anticancer agents include 18 candidates, 6 of which are in the late development stages [8].

**Figure 1:**
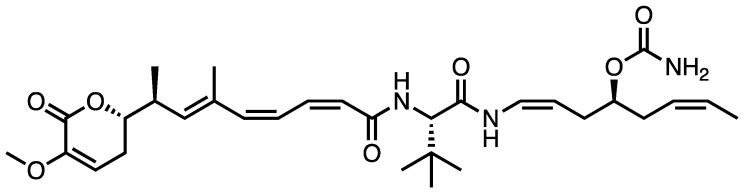
Structure of plocabulin.

Plocabulin (PM060184, C31H45N3O7) (Figure 1) is a polyketide isolated from the Madagascar marine sponge *Lithoplocamia lithistoides* [9,10]. Its antitumor properties have been evaluated in preclinical studies during last decade. Moreover, plocabulin undergoes phase I and phase II clinical trials in patients with advanced solid tumors. This review provides a critical overview of the anticancer activity of plocabulin, through the analysis of its key mechanisms of action, with special emphasis on its ability to counteract angiogenesis and tumor progression. The latest advances in its clinical development as an anticancer agent will also be discussed.

## 2. Antitubulin Activity

Microtubules still represent an excellent target for anticancer therapy due to their involvement in mitosis regulation [11,12]. The success of mitosis requires highly dynamic microtubules in the spindle. Polymerization dynamics are responsible for microtubules’ main biological function. For instance, during mitosis, microtubule dynamics regulate the proper attachment of chromosomes to the spindle, their alignment at metaphase and separation at anaphase. Thus, they are critical for the accurate segregation of chromosomes between the two daughter cells [13]. Agents inducing a sustained mitotic arrest at the metaphase/anaphase transition trigger apoptotic cell death. Microtubule-targeting agents are very successful in both hematopoietic and solid tumors, taking advantage of the rapid proliferation of cancer cells that more frequently pass through the stage of vulnerability to mitotic poisons than normal cells [11]. Natural vinca alkaloids and paclitaxel are two of the most-used microtubules targeting agents of terrestrial origin, but the sea also provides promising compounds, such as eribulin, dolastatins, and cryptophycins, whose anticancer activity is mainly imputable to their tubulin-binding ability [14].

Plocabulin belongs to a new family of marine microtubule-binding agents, showing different mechanisms of action compared to the other antitubulin marine derivatives [10,15]. Plocabulin binds with high affinity to tubulin dimers at the β-tubulin plus end and targets the longitudinal associations between tubulin dimers, but in a different site compared to vinblastine and the other marine tubulin-binding agents. In particular, plocabulin induces only weak associations of αβ-tubulin dimers compared to eribulin, which mainly inhibits tubulin polymerization [9]. Eribulin binds the vinca alkaloid domain at the interface between tubulin dimers and or/microtubules ends, resulting in the inhibition of microtubule growth but not in its shortening [16]. Differing from eribulin, plocabulin suppresses microtubule shortening and growing to a similar extent [17], affecting cells in both interphase and mitosis. Plocabulin’s effects on dynamic instability are also different to those of cryptophycin-52, which is more a potent inhibitor of microtubule shortening than an inhibitor of microtubule growth [18]. Later studies investigated the formation of ligand–tubulin complexes of plocabulin through X-ray and crystallography [19]. The results showed that plocabulin binds to the same site of the microtubule-destabilizing agents maytansine, a macrolide of terrestrial origin, and rhizoxin, an antimitotic agent of bacterial origin. The structures revealed a common pharmacophore for the three ligands and a mechanism for the inhibition of microtubules’ assembly that is different from that of the main clinically relevant agents that interfere with the binding site of vinblastine [19]. A very recent study provides further insight into the ligand–protein interactions responsible for the high affinity of plocabulin towards its binding pocket and a better understanding of the conformation and energetic effects arising from its association with the longitudinal interaction between tubulin dimers [20]. The atomistic molecular dynamics simulation and molecular mechanics with a generalized born and surface area solvation (MM/GBSA), a popular method to calculate the free energy of the binding of ligands to proteins, were used. They reveal that the tight tubulin–plocabulin binding was favored by the addition of a second tubulin dimer in longitudinal arrangement. These findings are useful for the future design of tubulin-binding agents and support the microtubule-destabilizing properties of plocabulin observed in different experimental models [20].

Six studies showed the antitumor activity of plocabulin (Table 1). Plocabulin binding with high affinity to β-tubulin alters microtubule dynamics and gives rise to the mitotic blockade and apoptosis of cancer cells. The studies in which plocabulin has been tested are presented according to the increasing complexity of the experimental strategy, from two-dimensional (2D) cell culture monolayers through three-dimensional (3D) models to xenograft animal models.

### 2.1. In Vitro Studies

Three different studies explored the anticancer activity of plocabulin in cancer cell lines cultured in 2D (Table 1). Pera and colleagues investigated its antiproliferative effects in 23 different tumor cell lines. Plocabulin showed considerably lower GI_50_ values than the well-known anticancer drugs vinblastine and paclitaxel in all tested cell lines [9]. Although all the three molecules target tubulin, the highest antiproliferative activity of plocabulin may be due to its exclusive binding site and the peculiar mechanism of action that leads to the inhibition of both microtubule polymerization and dynamics, as previously described. Although all GI_50_ are in the nanomolar range, the values are quite different depending on the tumor cell line. This suggests a different antiproliferative activity of plocabulin depending on the type and characteristics of the tumor.

It is well-known that the overexpression of the efflux pump P-glycoprotein (P-gp) generally correlates with chemoresistance, including resistance to tubulin-binding agents [25]. Interestingly, plocabulin inhibited the cell growth of three different P-gp overexpressing cell lines: the ovarian cancer cells IGROV-1, resistant to etoposide, and the ovarian A2780 and colon LoVo cancer cells, both resistant to doxorubicin. Although these cell lines were less sensitive to the antiproliferative effect of plocabulin compared to the parental cells (IGROV-1/ET: 10-fold, A2780/Dox: 6.8-fold and LoVo/Dox: 50-fold), the recorded GI_50_ were still in the nanomolar range (Table 1) and were considerably lower than that one of the positive controls, vinblastine (GI_50_ > 1300 nM in all three P-gp overexpressing cell lines) or paclitaxel (GI_50_ > 117 nM in all three P-gp overexpressing cell lines) [9,17]. Moreover, Martínez-Díez and colleagues demonstrated that plocabulin exhibited antiproliferative effects that directly correlated with its binding affinity to tubulin in lung A549 tumor cells [17]. In the same cell model, plocabulin favored the appearance of chromosome misaggregation and aberrant mitotic spindle, inducing G2/M cell-cycle arrest in a concentration-dependent fashion. These events envisaged the appearance of multipolar mitosis and lagging chromosomes at the metaphase plate, correlating with prometaphase arrest and cell death [17]. In the same study, the authors performed a wound-healing assay in A549 cells. They observed that the marine sponge product blocked the migration of cancer cells, highlighting its potential in the later stages of cancer development. The blockage of cell migration recorded in the wound-healing assay was probably due to the direct inhibition of cell migration by plocabulin, not just mediated by its antiproliferative effects.

Plocabulin showed cytotoxic activity towards ovarian cancer cell lines with different sensitivities to cisplatin treatment [21], including the very resistant cells PEA1, PEA2, PEO4, OV866(2), with IC_50_ in the low nM range (Table 1). Thus, plocabulin may be a promising treatment in cells resistant to conventional chemotherapeutic regimen. In the same panel of high-grade serous ovarian carcinoma cell lines, the anticancer effects of plocabulin were assessed in 3D spheroids [21]. Three-dimensi0onal cell cultures are used in cancer research as a bridge between in vitro and in vivo experimental models. Indeed, it is well-known that, compared to 3D cells, 2D cells are more susceptible to the anticancer activity of drugs. The lower diffusion gradient of the drug through the structure of the spheroid and the different distribution of oxygen, nutrients, and catabolites compared to the monolayer cultures reduce drug response in 3D cells [26]. Thus, it is not surprising that plocabulin only remained cytotoxic in the low nanomolar range in 3 of the 11 cell lines in the spheroid model (Table 1), even if the study tested plocabulin activity of up to 10 nM and not at higher concentrations [21]. The anti-invasion and anti-migration capacity of plocabulin was tested in both 2D and 3D models. Plocabulin reduced both transwell cell migration and invasion in PEO14, OV866(2) and PEA2 monolayer cells, while only a weak effect was recorded in OV866(2) spheroids [21]. This is the first evidence supporting the anti-migratory properties of plocabulin in 3D models, and requires further investigation. Moreover, the same study explored the combination of plocabulin with conventional chemotherapeutic drugs commonly used for the treatment of high-grade serous ovarian cancer, i.e., cisplatin, gentamicine, doxorubicin, trabectedin. None of the tested combinations showed synergistic or additive effects using the robust and restrictive tool SynergyFinder Plus score [21].

Organoids and spheroids are two different 3D models [27]. Organoids genetically and histologically resemble the original tumor from which they are derived, whereas spheroids are generally spherical cellular units, which are less complex compared to organoids when mimicking tumor organization [27]. Plocabulin anticancer activity was assessed in colorectal cancer organoids from three male patients (aged 81, 83 and 86 years) [22]. Samples were characterized for their mutation pathway and were treated with increasing concentrations of plocabulin for four days. Similar efficacies were observed in terms of IC_50_ among the three patients’ organoids (Table 1). Furthermore, the cytotoxic activity of plocabulin was compared to that of SN38, the active metabolite of the topoisomerase inhibitor irinotecan. The results showed that the cytotoxicity of plocabulin was one order of magnitude higher than that one of SN38 [22]. The results depict plocabulin as a promising treatment strategy, although only in three different types of colon cancer organoid, based on a personalized anticancer approach that considers the tumor mutation pathway.

### 2.2. In Vivo Studies

The xenograft model is an excellent experimental approach for predicting drug response in human tumors [28]. The anticancer effects of plocabulin were reported in six subcutaneous human-derived xenografted tumors (MDA-MB-231, HCT-116, HGC-27, H-460, 22RV1 and Caki-1) in female athymic nu/nu mice models. The curves in tumor growth after intravenous (i.v.) administration of 16 mg/kg of plocabulin on days 0, 7 and 14 showed concordant tumor growth inhibition (Table 1). Moreover, plocabulin’s ability to overcome the P-gp overexpression-mediated resistance, previously demonstrated in in vitro models, was confirmed in the xenograft model of colon cancer resistant to doxorubicin (LoVo/Dox) [17].

The anticancer effects of plocabulin were later investigated in patient-derived xenograft models of gastrointestinal stromal tumor (GIST). The standard treatment of this kind of tumor is with tyrosine kinase inhibitors such as imatinib, which contrast the gain-of-function mutations in the KIT oncogene, responsible for the uncontrolled cell proliferation [29]. However, resistance to tyrosine kinase inhibitors often occurs in patients with inoperable and metastatic GIST, mainly caused by secondary mutations of these kinases [30]. Thus, new therapies that do not target the driver oncogenic kinases are an urgent need. Plocabulin was i.v.-administered at 16 mg/kg once every week for 22 days in six-week-old, female, athymic Rj:NMRI-Foxn1 nu/nu mice xenografted by the bilateral implantation of a human GIST tumor fragment of three patients [23]. The tumors were characterized by a different mutation status for KIT and a consequent different sensitivity to imatinib therapy (UZLX-GIST3KIT 11 harbors KIT mutations in exon 11 and is sensitive to imatinib; UZLX-GIST9FKIT 11+17 harbors mutations in exons 11 and 17 and is resistant to imatinib; UZLX-GIST2BFKIT 9 harbors mutations in exon 9 and is resistant to imatinib (Table 1)). Plocabulin provoked significant tumor shrinkage in UZLX-GIST3KIT 11 and UZLX-GIST9FKIT 11+17, while its effect was reduced in UZLX-GIST2BFKIT 9. This difference may be due to the lower generation of tumors in the UZLX-GIST2BFKIT 9 xenograft model, which does not allow for a significant difference in terms of tumor shrinkage after plocabulin administration [23]. Treatment with polyketide induced extensive necrosis in the tumor tissue of all three types of tumors, leaving a small rim of viable cells at the periphery of the tumor. The extensive necrosis precluded further histological analyses of the samples in some cases. Furthermore, it was demonstrated that plocabulin did not affect the KIT signaling due to its different mechanism of action as a tubulin-binding inhibitor [23]. Taken together, these results provide a preclinical rationale for the investigation of the association between plocabulin and tyrosine kinase inhibitors in GIST. Thanks to their different mechanisms of action, plocabulin and tyrosine kinase inhibitors may counteract GIST development and the onset of resistance. Furthermore, the anticancer effects of plocabulin on GIST may unhinge the hypothesis that these tumors are clinically resistant to the non-tyrosine kinase inhibitor cytotoxic chemotherapeutic approach [31].

Soft-tissue sarcomas are a group of rare mesenchymal cancers characterized by their poor prognosis. Thus, the identification of new therapeutic strategies is impelling. Doxorubicin has represented the first line of therapy for advanced soft-tissue sarcoma for 40 years, despite providing a modest response rate of around 15%. Moreover, the combination with other cytotoxic drugs does not improve clinical outcome and overall survival [32]. Wang and colleagues investigated plocabulin efficacy in seven patient-derived xenograft models, which were generated by the engraftment of sarcoma tumor fragments directly into nude mice [24]. Five distinct histological subtypes of soft tissue sarcoma were analyzed: dedifferentiated liposarcoma, leiomyosarcoma, undifferentiated sarcoma, intimal sarcoma, and capicua transcriptional repressor (CIC)-rearranged sarcoma. The volumetric analysis of tumor after plocabulin treatment revealed tumor volume control in five histological subtypes (Table 1). In particular, volume stabilization was recorded in dedifferentiated liposarcoma and intimal sarcoma, while tumor regression was observed in leiomyosarcoma, CIC-rearranged sarcoma, and undifferentiated sarcoma models. The volume control of the tumor was more efficient under plocabulin treatment compared to doxorubicin-treated mice. Moreover, in the three most responsive sarcoma subtypes, extensive central necrosis and tumor regression were recorded, whereas in the two less responsive subtypes, dedifferentiated liposarcoma and intimal sarcoma, zonal necrosis was detected [24]. Of note, as previously observed in xenografted GIST models and sarcoma tumors, there was a thin viable rim of tumor cells at the periphery of the lesion, possibly leading to cell proliferation and metastasis. This is a typical feature of solid tumors that undergo treatment with vascular-disrupting agents [33], as will be explained in the next paragraph. In the external area, tumors can take advantage of nearby vessels located in the surrounding normal tissue; thus, they can make use of nutrients and oxygen to grow. It is not possible to estimate which type of sarcoma will be more responsive to plocabulin from the results obtained for the different subtypes. However, it is possible to partially explain the reason that dedifferentiated liposarcoma was one of the least responsive to plocabulin treatment. Dedifferentiated sarcoma is commonly characterized by an overexpressed chromosome region, 12q24, containing the gene EIF2B1, which encodes for the eukaryotic translation initiation factor 2B (eIF2Bα) [34]. This protein has been shown to be involved in an in vitro mechanism of resistance towards plocabulin [35]. Indeed, a gain-of-function mutation in the α subunit of this protein leads to hyperactivation of the integrated stress response of eukaryotic cells to stress stimuli, conferring resistance to plocabulin. This was recently demonstrated using a fungal model, whose microtubules are similar to the mammalian ones [35]. The identification of specific markers that are possibly involved in the mechanism accounting for plocabulin resistance can help to predict tumor sensitivity to this anticancer treatment.

## 3. Antiangiogenic Activity

Tumor vasculature is essential in the regulation of tumor growth, invasion, and metastasis. Thus, it is currently accepted that the inhibition of angiogenesis is an effective strategy to counteract cancer development and dissemination [36,37]. Many microtubule-binding agents have relevant antiangiogenic and vascular-disrupting properties [38]. The vascular-disrupting action is mainly due to a direct antiproliferative effect on endothelial cells, the inhibition of their migration and tube formation. Indeed, actin and microtubule cytoskeletons play a key role in the organization of a three-dimensional vessel structure, regulating the maintenance of endothelial cell shape and their proliferation. The antiangiogenic effects of tubulin-binding agents have been observed both in vitro and in vivo, where they induce a rapid vascular collapse [39]. Accordingly, the microtubules stabilizer docetaxel retained its antitumor activity in an ovarian cancer xenograft model docetaxel-resistant thanks to its antiangiogenic actions [40]. Notably, there are data suggesting that the effect of microtubule-binding agents on endothelial cells and the resulting reduction in blood flow are higher in tumor tissues compared to normal tissues, potentially contributing to their selective anticancer efficacy [38]. These observations prompt the development of new microtubule-binding drugs with antiangiogenic activity.

In this context, plocabulin showed relevant antiangiogenic properties. Its ability to work as a vascular disruptor was due to the inhibition of microtubule dynamics in endothelial cells, which leads to alterations in the tumor’s vascular architecture [41]. Interestingly, plocabulin did not induce significant changes in the angiogenic-related proteins in endothelial or tumor cancer cells [41]. This represents an advantage compared to other cytotoxic drugs, which increase the secretion of growth and pro-survival factors, possibly reverting the antiangiogenic activity [36]. Plocabulin inhibited the in vitro migration and invasion of human umbilical vein endothelial cells (HUVEC). Moreover, it interfered with HUVEC’s ability to induce the formation of a 3D capillary-like network, as well as disrupting pre-existing vessels [41]. The subsequent collapse of the endothelial tubular network occurred in a concentration-dependent fashion (0.01–1 nM) and was recorded at concentrations lower than those affecting cell survival. Indeed, at 1 nM, cell viability was 100%. These results were confirmed in nude mice xenograft models of lung adenocarcinoma (xenografted with NCI-H460) and breast carcinoma (xenografted with MDA-MB-231) after i.v. administration of three consecutive weekly doses of 0.08, 8 or 16 mg/kg/day of plocabulin [41]. The in vivo antiangiogenic effects were characterized by a significant reduction in vascular volume and the shutdown of new vessels’ formation, accompanied by extensive necrosis of the tumor tissue.

A more recent in vivo study performed by Wang et al. [22] confirmed the strong vascular-disruptive anticancer effects of plocabulin in patient-derived xenograft models of GIST [23]. Mice engrafted with tumors were treated with 16 mg/kg of plocabulin. The antiangiogenic effects were recorded through the analysis of CD31 expression, which showed a decrease in the total vascular area containing shorter and smaller microvessels with a collapsed lumen compared to mice treated with the vehicle. Of note, antiangiogenic activity was observed in absence of effects on proliferation or apoptosis. The histological outcome of this vascular-disrupting effect was central necrosis [23]. The dose of 16 mg/kg used in the study corresponds to a human equivalent dose of 1.3 mg/kg or 48 mg/m^2^ [42], which is much higher than the doses used in the current clinical trials (i.e., from 9.3 mg/m^2^ to 11.6 mg/m^2^) (NTC03427268; Eudra CT 2015-002395-24). Thus, it is not possible to predict whether the dose tested in the study by Wang et al. is physiologically relevant and safe in humans. However, the extent of the vasculature damage was so severe that it is possible to speculate that it could be induced at even lower doses than 16 mg/kg, especially if plocabulin is administered with a tyrosine kinase inhibitor in a combination therapeutic regimen.

The damage and shutdown of tumor vasculature recorded in GIST-xenografted mice was also observed in sarcoma-xenografted mice treated with 16 mg/kg of plocabulin, with the exception of the dedifferentiated liposarcoma type [24]. Tumor vasculature after treatment with plocabulin was analyzed through the immunohistochemical analysis of CD31, as for GIST tumors. The staining of CD31 was performed using a primary antibody that targets the murine epitope without cross-reaction with the human counterpart. This could represent a limit of the study, because microvessels of murine origin might be more sensitive to the antiangiogenic effects of plocabulin than human ones. Further investigations are needed to clarify the anti-angiogenic efficacy of plocabulin in humans.

Anti-angiogenic effects can represent a double-edged sword because a preserved tumor vasculature is necessary for efficient anticancer drug delivery to the tumor tissue [43]. On the other hand, the inhibition of angiogenesis counteracts the processes of invasion and metastasis [44]. An interesting property of plocabulin is that it exhibits both cytotoxic and antiangiogenic activities and this can result in a potent anticancer effect in the early and later stages of cancer progression.

## 4. Clinical Studies

In vitro and in vivo studies have shown a multi-target effect of plocabulin, mediated by the inhibition of microtubule polymerization and dynamics and antiangiogenic effects [45,46]. The different and complementary antitumor activity can be an interesting rationale for the clinical development of plocabulin in the monotherapy of advanced cancers resistant to cancer chemotherapy. To date, some clinical trials have investigated the efficacy and the safety profile of plocabulin in patients with advanced cancers (Table 2).

A first dose-escalation, phase I study investigated the dose-limiting toxicities (DLTs), the maximum tolerated dose (MTD) and the recommended dose (RD) for phase II trials of plocabulin in 44 patients (23 males and 21 females, median age 53 years, range 22–72 years) with advanced/metastatic solid tumors (Table 2) [47]. The most common primary tumors included in the trial were colorectal carcinoma (n. 11), breast carcinoma (n. 5), cervix carcinoma (n. 5), non-small cell lung cancer (NSCLC, n. 5), and others (n. 18). The enrolled patients had previously received at least one standard chemotherapeutic treatment for advanced cancer disease, including platinum compounds, pyrimidine analogues, anthracyclines, vinca alkaloid or biological therapy.

The pharmacokinetic profile investigated during the first-cycle treatment showed a half-life of plocabulin of ~4 h and a wide diffusion to peripheral tissues [47]. In the dose-escalation study, plocabulin was administered i.v. over 10 min at a starting dose of 1.3 mg/m^2^ on day (D) 1, D8 and D15 every four weeks. The onset of grade 3 peripheral sensory neuropathy in two patients was used to establish the MTD at 14.5 mg/m^2^. During the study, the different administered doses of plocabulin provoked milder grade (1–2) peripheral sensory neuropathy and fatigue in 71% and 60% of patients, respectively. These toxicities were responsible for dose-omission, delays, or interruptions in the treatment. Other DLTs included cardiac effects and tumor lysis syndrome, whereas the myelosuppression was transient and manageable. Among the DLTs, peripheral neuropathy is an expected side effect, as it is well-known that the microtubule inhibitors can cause chemotherapy-induced peripheral neuropathy (CIPN), which is prevalent and severe and results in dose interruptions, sub-therapeutic dosing, or discontinued therapy [48]. CIPN is a predominantly sensory neuropathy that may be accompanied by motor and autonomic changes [49,50]. In this context, the abdominal pain observed in this trial (30% of cases) might be related to autonomic peripheral neuropathy, while myalgia (27%) might be a symptom of motor peripheral neuropathy [47]. This trial failed in the identification of the phase II RD due to dose delays and omission, which resulted in a too-low compliance at the 12 mg/m^2^ expansion cohort [47]. In addition, the preliminary efficacy of plocabulin was investigated in thirty-six enrolled patients. The administration of 12 mg/m^2^ induced a partial response in a patient with cervix carcinoma, with 44% of tumor shrinkage, and in a patient with metastatic NSCLC, with 36% of tumor shrinkage. Moreover, disease stabilization for >3 months was observed in 10 patients (4 colorectal carcinoma, 2 GIST, 1 breast, 1 cervix, 1 thymus, 1 parotic carcinoma) [47]. Since all patients were previously treated with a median of 4 conventional chemotherapy lines, the results were considered promising and the antitumor activity of plocabulin was evaluated in further trials.

Another phase I, multicenter, open-label trial was performed to determine the MTD and the RD, and to assess the toxicity and antitumor activity of plocabulin in patients with advanced solid tumors (Table 2). Sixty patients were enrolled in the study and plocabulin was administered i.v. over 10 min at a starting dose of 4 mg/m^2^ on three consecutive days (on D 1–3 and 15–17) every 28 days. The study is completed, but data on the safety and anticancer activity of plocabulin are not yet available (NCT01299636), with the last update on the trial posted in 2015.

A phase II, randomized control study investigated the efficacy of plocabulin in terms of progression-free survival at 4 months in 22 women with advanced, hormone receptor positive, and human epidermal growth factor receptor 2 negative breast cancer (Eudra CT 2015-002395-24) (Table 2). All the patients were previously treated with anthracyclines and taxanes. Plocabulin was administered i.v. at a dose of 9.3 mg/m^2^ on D1 and D8 every three weeks. No patient completed the study: the main reasons for this included disease progression (12 cases) and refusal of treatment (6 cases). The problems raising during the study and the small number of enrolled patients precluded the assessment of plocabulin antitumor efficacy.

Applying the same dose and schedule treatment, the efficacy of plocabulin has been evaluated in another phase II study in 32 patients with advanced colorectal cancer after standard therapy (fluoropyrimidine, irinotecan, and oxaliplatin) (Table 2). The progression-free survival rate at 12 weeks was the primary endpoint of the study (NCT03427268). However, the study did not provide conclusive information on the potential antitumor activity of plocabulin. Only 20.7% of the participants reached the primary endpoint and a high all-cause mortality occurred during the study (53%). Regarding the safety profile, 6 patients developed serious adverse events, and side effects were observed in the entire study population. The most common toxicities included asthenia (73%), abdominal pain (66%), constipation (63%), and paresthesia (46%). Following plocabulin treatment, no complete response or partial responses were observed in the study population, and 13 patients reached progressive disease. Promising initial evidence could be the disease stabilization for >3 months observed in 7 patients (24,1% of participants). These findings agree with the results of the trials discussed above and indicate the therapeutic potential of plocabulin to counteract disease progression in patients with advanced cancer that is resistant to traditional chemotherapy [47].

The preclinical studies described above showed that the antiangiogenic and vasodisruptive effects of plocabulin were concentration-dependent and occurred at a concentration lower than dose-impairing proliferation or cell survival [23,41]. It can be assumed that plocabulin can be administered at a lower dose when used in combination therapy with other anticancer drugs. To our knowledge, only one phase I clinical trial as investigated escalating doses of plocabulin in combination with gemcitabine, in 57 patients (42 females, 15 males) with advanced solid tumors (NCT02533674). The results of this clinical trial are still under analysis, and it is not possible to draw any conclusion regarding the anticancer efficacy of the combination of the two drugs. Thus, although four clinical studies were performed, the available evidence does not allow for an exhaustive comprehension of the safety and clinical benefits of plocabulin, either alone or in combination therapy.

## 5. Conclusions and Future Perspectives

**Figure 2:**
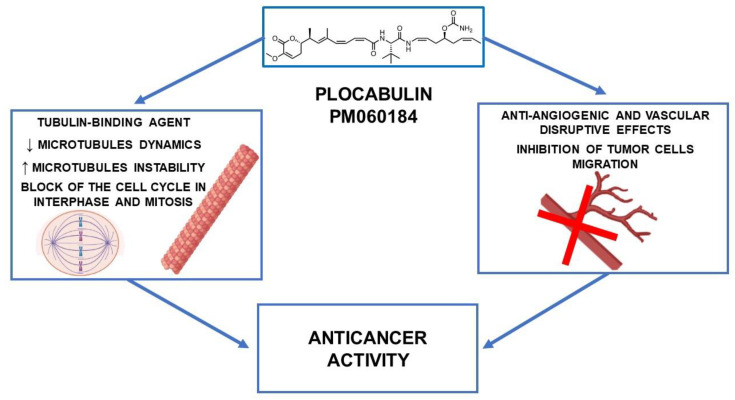
Anticancer activity of plocabulin (with the use of BioRender.com, accessed on: 7 December 2022).

Plocabulin is a promising anticancer agent that inhibits tubulin polymerization by binding the tubulin dimer end with one of the highest known affinities. The effects of that interaction result in alterations in the dynamic instability of the microtubules, affecting cells in both interphase and mitosis, and leading to cell growth inhibition and cell death. The anticancer activity of plocabulin has been demonstrated both in vitro and in vivo. Of note, anticancer activity was also observed in tumor cells resistant to traditional chemotherapy agents (i.e., cisplatin or doxorubicin). Besides, the inhibition of microtubule dynamics in endothelial cells by plocabulin leads to alterations in tumor vascular architecture and plocabulin’s antiangiogenic effects (Figure 2).

Despite the convincing evidence in preclinical studies, the clinical anticancer efficacy of plocabulin in advanced cancer treatment is unclear. Indeed, not all the results of clinical trials are already available and a limited number of patients in the published trials completed the treatment cycle with plocabulin. However, the clinical evidence mainly showed stabilization of cancer progression up to 3 months after plocabulin treatment in colorectal, breast, thymic, and GIST tumors, among others. This is an encouraging outcome, mainly because the enrolled patients were previously and unsuccessfully treated with different chemotherapeutic drugs. The planning of future clinical studies should consider the different anticancer activity of plocabulin, according to the type and histological characteristics of the tumor emerging from in vitro and in vivo studies. Only the enrollment of a representative selection of patients with solid advanced cancer, stratified according to the aforementioned characteristics, could help us to understand if there is a preferential, histologically driven response.

The definition of the RD is another critical point for the clinical development of plocabulin. At the estimated MTD of plocabulin, several DLTs were recorded, including the peripheral sensory neuropathy typically associated with tubulin-binding agents. Preclinical studies showed the possibility of obtaining vascular-disruptive effects of plocabulin at lower doses than the cytotoxic ones. This evidence prompted us to investigate the use of a low dose of plocabulin as an antiangiogenic agent to improve the anticancer activity of other cytotoxic drugs. To date, neither preclinical studies nor clinical trials have provided sufficient knowledge on the combination therapeutic potential of plocabulin with standard anticancer drugs, and, in our opinion, this approach deserves further research.

In recent years, the supply of marine products was discussed from the ethical and legislative perspective, and the Nagoya protocol, entered into force in 2014, represents one of the first results of this reflection [46]. The production of the polyketide by an efficient synthetic strategy has overcome the supply problem and prompted clinical studies to define its therapeutic potential in cancer patients.

The passage of plocabulin from seaside to bedside is still at its beginning. Further efforts are necessary for the translation from experimental evidence to possible clinical uses for plocabulin-based strategies in the later stages of cancer progression.

## Figures and Tables

**Table 1:** In vitro and in vivo studies on the anticancer activity of plocabulin.

Experimental Models	Plocabulin: Concentrations/Doses and Time of Treatment	IC_50_ or GI_50_ (nM)		Association with Other Anticancer Drugs	Mechanisms of Action	References
Human prostate (PC3, 22RV1), pancreas (PANC-1, MiaPaCa-2), ovary (IGROV-1, A2780), lung (NCI-H460, NCI-H23, A549), liver (SK-HEP-1, HEPG2), leukemia (MOLT4, K562), kidney (RXF393, CAKI-1), stomach (HS746T, HGC-27), colon (LoVo, HT29, HCT-116), and breast (MDA-MB-231, MCF-7, BT-474) cancer cell lines	Range of concentrations tested not indicated 72 h	GI_50_ PC3, 0.114 22RV1, 0.0636 PANC-1, 0.0997 MiaPaCa-2, 0.145 IGROV-1, 0.0429 A2780, 0.152 NCI-H460, 0.101 NCI-H23, 0.129 A549, 0.0892 SK-HEP-1, 0.752 HEPG2, 2.76 MOLT4, 0.102 K562, 0.151 RXF393, 0.0420 CAKI-1, 0.525 HS746T, 2.10 HGC-27, 0.0659 LoVo, 0.146 HT29, 0.0403 HCT-116, 4.68 MDA-MB-231, 0.0909 MCF-7, 4.07 BT-474, 0.054			↑ cellular microtubules disruption ↓ mitosis ↓ cellular proliferation	[9]
Human ovarian cancer cell lines cultured in 2D or in 3D spheroids (PEA1, PEA2, PEO1, PEO4, PEO6, PEO14, PEO23, PEO16, OVCAR-3, 59M, OV866(2), TOV3041G)	Up to 10 nM 72 h	IC_50_ 2D PEA1, 0.07 PEA2, 0.23 PEO1, 0.03 PEO4, 0.05 PEO6, 0.37 PEO14, >10 PEO23, 0.35 PEO16, 0.30 OVCAR-3, 0.03 OV866(2), 0.08 TOV3041G, 0.07 59M, 1.15	IC_50_ 3D >10 >10 >10 0.16 0.24 >10 >10 0.05 >10 >10 >10 >10	No synergistic or additive effects with cisplatin, gemcitabine, doxorubicin, trabectedin	↑ depolymerizing effect on microtubules ↓ invasion (PEA1, PEA2, PEO14 and OV866(2) in 2D) ↓ migration (PEA2, PEO14 and OV866(2) in 2D and OV866(2) also in 3D spheroids)	[21]
Colorectal cancer patient-derived tumor organoids	Up to 5 nM 96 h	IC_50_ Patient#3, 1.1 Patient#4, 0.9 Patient#29, 0.7			↓ cell viability of colorectal cancer organoids	[22]
Human ovarian cancer (IGROV-1, IGROV/ET, A2780, A2780/Dox), human colon cancer (LoVo, LoVo/Dox) cell linesXenografted (MDA-MB-231, HCT-116, HGC-27, H-460, 22RV1 and Caki-1) female athymic nu/nu mice	Range of concentrations tested not indicated 72 h 16 mg/kg i.v. (0, 7, 14 day)	GI_50_ IGROV-1, 0.4 IGROV-1/ET, 4.0 A2780, 2.5 A2780/Dox, 17 LoVo, 0.1 Lovo/Dox, 5.0			↓ tubulin polymerization, alterations in the dynamic instability of microtubules, and blockage of the cell cycle in both interphase and mitosis ↓ microtubules’ shortening and growing to a similar extent ↓ tumor growth	[17]
3 patient-derived xenografted nude mice of GIST characterized by different GIST mutations (UZLX-GIST3KIT 11 harbored KIT mutation in exon 11; UZLX-GIST9FKIT 11+17 harbored mutations in exons 11 and 17; UZLX-GIST2BFKIT 9 harbored mutation in exon 9)	16 mg/kg i.v.once a week for 22 days				↓ tumor growth ↑ tumor necrosis ↓ tumor vasculature	[23]
7 patient-derived xenografted nude mice of sarcoma (dedifferentiated liposarcoma, leiomyosarcoma, undifferentiated sarcoma, intimal sarcoma, and CIC-rearranged sarcoma)	16 mg/kg i.v. once a week for 22 days				↓ tumor growth Tumor stabilization in dedifferentiated liposarcoma and intimal sarcoma Tumor regression in leiomyosarcoma, CIC-rearranged sarcoma, and undifferentiated sarcoma models ↑ tumor necrosis ↓ tumor vasculature	[24]

↑: increase; ↓: decrease; IC_50_: half maximal inhibitory concentration; GI_50_: concentration that inhibits 50% of cell growth; i.v.: intravenously; GIST: gastrointestinal stromal tumor; IGROV-1/ET: IGROV-1 cells resistant to etoposide, overexpressing P-gp; A2780/Dox: A2780 cells resistant to doxorubicin, overexpressing P-gp; LoVo/Dox: LoVo cells resistant to doxorubicin, overexpressing P-gp; CIC-rearranged sarcoma: capicua transcriptional repressor-rearranged sarcoma.

**Table 2:** Clinical studies on the anticancer activity of plocabulin.

Phase	Population	Intervention	Key Outcome(s)	Status and/or Key Results	Reference or Clinical Trial Identification Number
I	Forty-four patients with advanced solid tumors (11 colorectal carcinoma, 5 breast carcinoma, 5 cervix carcinoma, 5 NSCLC, others ^a^)	Plocabulin (i.v.), starting dose: 1.3 mg/m^2^ administered on D1, D8 and D15 every four weeks	DLTs, MTD, RD	MTD = 14.5 mg/m^2^, 2/2 patients with DLTs (grade 3 peripheral sensory neuropathy)	[47]
I	Sixty patients with advanced solid tumors	Plocabulin (i.v.), starting dose: 4 mg/m^2^ administered on D1–3 and D15–17 every 28 days	MTD, RD	Completed. Results not yet available.	NCT01299636
I	Fifty-seven patients with advanced solid tumors (18 NSCLC, 13 endometrial or cervical cancer, 13 epithelial cancer, 4 breast cancer, others ^b^)	Plocabulin (6–10.5 mg/m^2^) and gemcitabine (800 or 1000 mg/m^2^)	DLTs, MTD, RD	9% of patients with DLTs (44 patients evaluated), all-cause mortality: 14.5% (55 patients evaluated), serious adverse events: 47.3% (55 patients evaluated)	NCT02533674
II	Twenty-two women with advanced, hormone receptor positive, HER2 negative breast cancer	Plocabulin 9.3 mg/m^2^ on D1 and D8 every three weeks	PFS rate at 4 months (primary endpoint), OS	PFS rate: 11.1% (18 patients evaluated), OS: 6.6 months (median value; 18 patients evaluated), serious adverse events: 19% (21 patients evaluated)	Eudra CT 2015-002395-24
II	Thirty-two patients with advanced colorectal cancer	Plocabulin 9.3 mg/m^2^ on D1 and D8 every three weeks	PFS rate at 3 months (primary endpoint), OS	PFS rate: 20.7% (29 patients evaluated), OS: not reached (29 patients evaluated), all-cause mortality: 53% (30 patients evaluated), serious adverse events: 20% (30 patients evaluated)	NCT03427268

D: day; DLTs: dose-limiting toxicities; HER2: human epidermal growth factor receptor; MTD: maximum tolerated dose; NSCLC: non-small cells lung cancer; OS: overall survival; PFS: progression free survival; RD: recommended dose. ^a^ 3 GIST, 3 pancreas adenocarcinoma, 3 soft-tissue sarcoma, 1 cholangiocarcinoma, 1 endometrium adenocarcinoma, 1 germinal testicular carcinoma, 1 head and neck carcinoma, 1 malignant melanoma, 1 ovarian adenocarcinoma, 1 parotid adenocystic carcinoma, 1 thymus carcinoma, 1 urothelial carcinoma. ^b^ 3 GIST, 3 head and neck cancer, 2 germ cell tumors, 1 adenocarcinoma or carcinoma of unknown primary site.
